# Fine structural characteristics of the chorionic microspheres on the egg surface of the orb web spider *Trichonephila clavata*

**DOI:** 10.1186/s42649-023-00087-4

**Published:** 2023-07-18

**Authors:** Seung-Min Lee, Myung-Jin Moon

**Affiliations:** Department of Biological Sciences, Dankook University, Cheonan, 31116 Korea

**Keywords:** Eggcase, Eggmass, Electron microscope, Overwintering, Oviposition

## Abstract

The eggs laid by the orb web spider *Trichonephila clavata* must overwinter in bitterly freezing and dry conditions before hatching, but there does not seem to be any protection like a compact silk case covering the entire eggmass. Instead, the surface of the eggmass is completely coated with a milky coating called chorionic microspheres (CM). Therefore, we investigated the fine structural characteristics of CM to demonstrate their ecological importance. Although the diameter of CM in outer eggmass exhibits a significant variation, the chorionic surface is coated with a single layer of CM, characterized by a consistent diameter of approximately 2.3 µm. The surface structure of aggregated CM shows short papillary projections demonstrating segmental adhesion of mucous components. CM is insoluble in water but partially soluble in anhydrous ethanol, and its spherical structure is completely decomposed by hexafluoroisopropanol (HFIP), a strong organic solvent. Since our fine structural observations clearly show that CM is not derived from vitellogenic or choriogenetic processes, the CM adhesive coatings during ovipositional process appears to be equivalent to cocoon silk for various protective functions in silken eggcase.

## Introduction

Maternal secretions can be observed on the egg surface of arthropods. The secretions have been identified in terrestrial arthropods, including dragonflies (Family: Libellulidae) (Miller 1987) and mites (Family: Acarididae) (Witaliński 1993), among others. In Megaloptera, there can be variations in the color of secretions depending on the species, and their physical characteristics can also range from being sticky to easily becoming powdered (Yu et al. 2022). On the other hand, in Araneae, rather than having an amorphous secretion structure, microspherical and crystalline units have been reported across the eggs of 21 families and 53 genera (Grim and Slobodchikoff 1978, 1982; Humphreys 1983, 1987).

The eggs of spiders, unlike that of other terrestrial arthropods, do not have a shell structure that could protect them during embryonic development (Schultz 1987). Instead, the outer membrane of their eggs is surrounded by two layers: the inner vitelline membrane and the outer chorion (Foelix 2011). The chorion contains chorionic microspheres (CM) of different diameters and densities that vary based on the species type (Grim and Slobodchikoff 1982). Nevertheless, the spherical morphology of CM has no significant correlation with the arachnid taxa (Humphrey 1983, 1987).

Grim and Slobodchikoff (1978) argued that CM reduce fungal infections by delaying water absorption. Austin and Anderson (1978) suggested that the CM were hydrophobic and therefore provide protection against moisture. Hadley (1978) confirmed the presence of a lipid layer impermeable to water on the egg surface. Witaliński and Žuwała (1981) suggested that the mucus-containing oviposition fluid coats the chorion, causing aggregation of eggs. Conti et al. (2015) suggested that the aggregation of the eggs restricts their dehydration, as they become tightly attached to each other. Despite the various functionalities proposed for CM, the observation of its fine morphology remains severely limited.

Most spiders wrap their eggs in cocoon silk derived from the tubuliform silk gland (Foelix 2011). The cocoon silk is known to protect the eggs from predators and parasites (Austin 1985), reduce the predator's perception of the background (Barrantes 2013), decrease desiccation during a long overwintering period (Hieber 1992), and regulate the temperature (Hieber 1985). Some species, such as Theridiidae and Lycosidae, build their cocoon as a compact “eggcase”, while others like *Trichonephila edulis* use a loose “eggpad” (Grim and Slobodchikoff 1982; Humphrey 1995). In this context, the density of silk is determined by whether the internal eggs are visible through it.

The golden orb web spider *T. clavata* is one of the most commonly species in Northeast Asia in late autumn. It endures the harsh Korean winter (up to approximately -18 °C) until hatching in the following spring, but lays eggs with in loose eggpads containing large amount of CM. Previously found only in Eurasia, this species was recently reported for the first time in the Americas (Hoebeke et al. 2015) and has expanded its range from southern United States as far as Oklahoma (Davis and Frick 2022).

We herein describe the fine structure of the chorion, attached with a massive amount of CM, of the orb-web spider, *Trichonephila clavata*, with aid of an electron microscope. To understand the role of CM on the spider ecophysiology, solubility CM in water and organic solvents was evaluated. This study further examined the abdomen of *T. clavata*, a sexually mature individual to determine the origin or source of the CM.

## Materials and methods

Adult females of the golden orb web spider, *Trichonephila clavata* were collected in a local area near the Dankook University, Chungnam, Korea. This species was moved to *Trichonephila* from the genus *Nephila* along with 10 other species (Kuntner et al. 2018).

All spiders were maintained under ambient conditions with natural lighting in enclosures comprising a wooden frame (height × length × width, 50 cm × 50 cm × 10 cm) with glass panels on the front and back, and fed insect larvae and water daily. After spawning, the spiders were released, and the eggmasses were stored in room temperature.

A shallow hole spot was cautiously made in center of the ellipsoidal eggmass using beauty scissors. The resulting crack was delicately widened along the shorter axis, dividing the mass into two pieces. Starting from the cut surface, inner eggs were carefully separated. After classifying inner eggs from the outer eggs with white coating piece, each egg was attached to a flexible cover glass (0.14 mm × 18 mm × 18 mm) (Marienfeld, Lauda-königshofen, Germany) and were treated for various solvent (distilled water, anhydrous ethanol, hexafluoroisopropanol; HFIP) conditions under different times (20 s, 1 h, 24 h). Considering the high volatility of HFIP, it rapidly evaporates upon exposure to air, and we estimate the processing time for this evaporation to be approximately 20 s.

Microscopic images were photographed using Motic digital imaging system (Motic Instruments Inc., Richmond, Canada) and Nikon Eclipse 80i microscope.

For scanning electron microscopic examination, the samples were mounted on stubs using double sided tape and were coated with platinum-palladium with a thickness of 20 nm, using an ion sputter coater (E-1030, Hitachi, Tokyo, Japan). Coated samples were observed with a field emission scanning electron microscopy (FESEM) (S-4300, Hitachi, Tokyo, Japan) with an accelerating voltage of 5–20 kV (Sun et al. 2021).

To analyze egg development in the maternal ovary, specimens were adequately anesthetized for 3 min with pure carbon dioxide. Then, specimens were slowly cooled down to 4℃ and dissected under light microscope in a drop of spider Ringer’s solution consisting of 160 mM NaCl, 7.5 mM KCl, 4 mM CaCl_2_, 1 mM MgCl_2_, 4 mM NaHCO_3_, 20 mM glucose, pH 7.4 (Moon and Tillinghast 2020). The ovary was gently removed. For transmission electron microscopy examination, the tissues were fixed in a mixture of 2% paraformaldehyde and 2.5% glutaraldehyde buffer with phosphate buffer.

Post-fixation was performed with 1% osmium tetroxide in the same buffer solution. They were dehydrated in graded concentrations of ethanol and propylene oxide, and embedded in EM-Bed 812 medium (Sun et al. 2023). Semithin sections stained with toluidine blue were used to examine the histology of the tissues. Ultrathin sections were obtained from a LKB ultramicrotome, and were double stained with uranyl acetate and lead citrate. The sections were examined with a transmission electron microscopy (TEM) (JEM 100 CX-II, JEOL, Tokyo, Japan) at 80 kV.

Statistical analyses were performed using SPSS 21 Statistics (IBM Korea, Seoul, South Korea) to determine frequencies of CM size variation by location. A *p* value of < 0.05 was considered statistically significant.

## Results

**Fig. 1:**
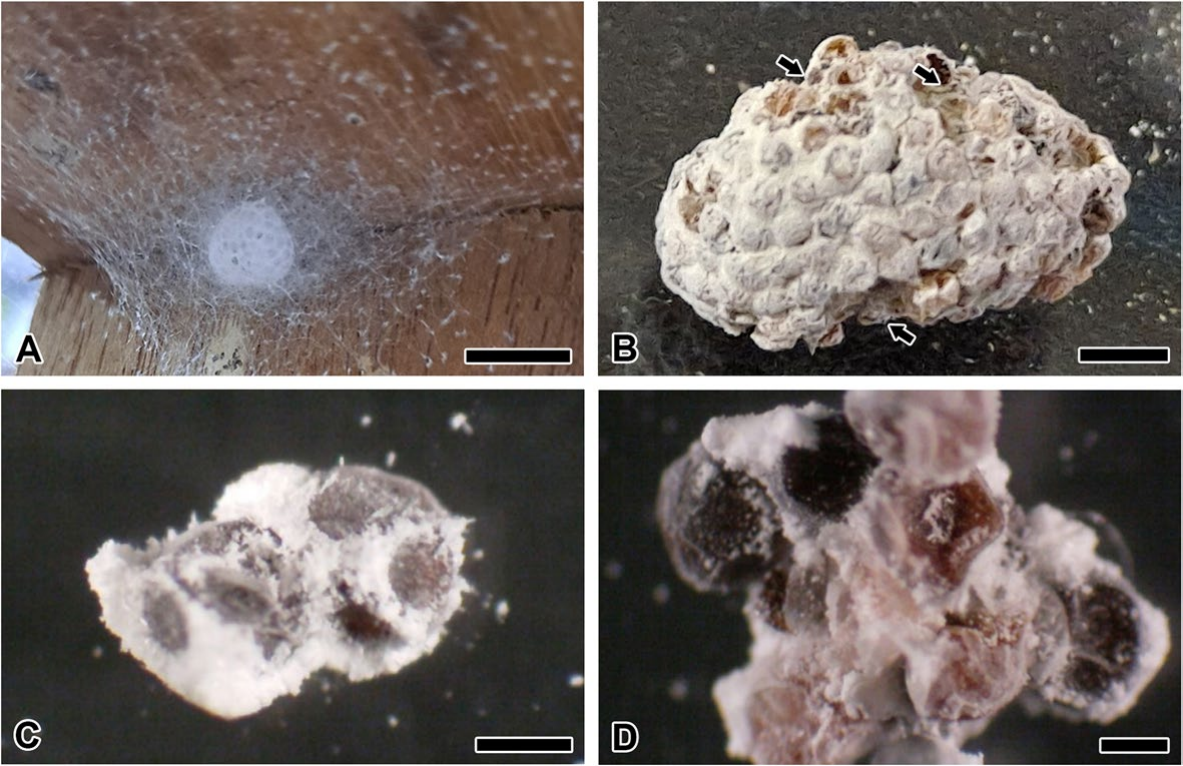
Photo micrographs of the eggmass of the spider *T. clavata*. **A** Randomly wrapped in loose silk, an eggmass is observed. **B** The surface of the eggmass is coated with a white milky secretion. Arrows indicate inner eggs, showing the difference compare with the outer coating. **C** Eggs form a mass by secretions and form lumps by filling the empty space inside. **D** The inner section of the eggmass reveals the chorion surface. Each scale bar indicates 1 cm (**A**), 2 mm (**B**) and 500 µm (**C**, **D**), respectively

The eggs are clustered in an ellipsoidal shape to form a mass (Fig. 1A). Following spawning, the eggmass is randomly wrapped with eggcase silk. The wrapping silk is characterized by an extensive enveloping of the eggmass. The eggpad is attached radially to the wooden frame by the pyriform (also called piriform) gland silk.

A thin coating comprising a milky substance surrounds the outer edge of the eggmass (Fig. 1B). This coating is prone to physical impact, leading to its fragmentation into particles. Eggcase silk passes through the inside of the coated surface. The empty space among eggmass is filled with the white lump (Fig. 1C). Unlike the outer coating surface, inner eggs have a low coating distribution, which allows the observation of the egg chorion (Fig. 1D). The egg is generally crumpled spherical and attached to each other.

**Fig. 2:**
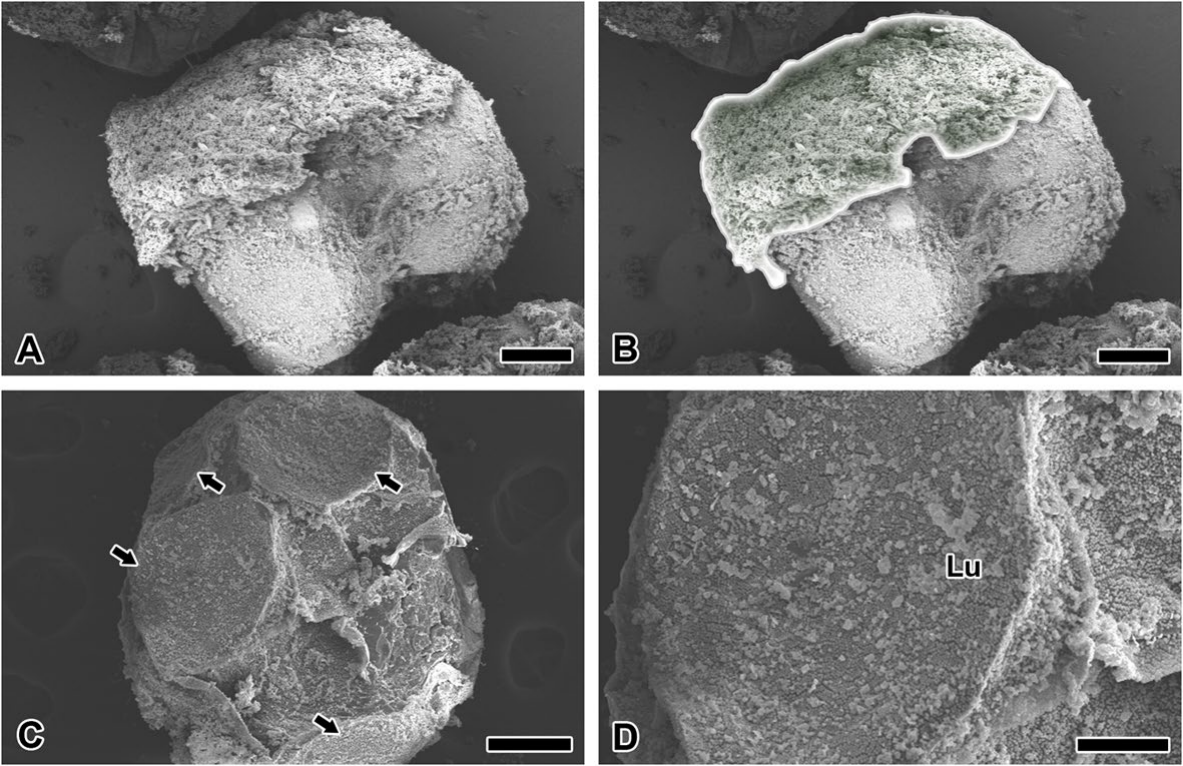
Eggmass surface and egg surface. **A** A thick milky white granular layer is formed on the outer surface of the eggshell, but not on the inner egg surface. **B** Outline-added Fig. 2A shows coating location. **C** Eggs inside the mass are characterized by a pressed surface (arrows) by the attachment surface with adjacent eggs. **D** Microspheres are also coated on the adhesive surface in the form of a homogeneous single layer. Lump of microspheres (Lu) are observed above the single layer. Each scale bar indicates 200 µm (**A**-**C**) and 50 µm **D**

The coating substance on the eggs were photographed with FESEM (Fig. 2A). A color effect was applied to distinguish the outer surface of the eggmass from the inner surface of the egg (Fig. 2B). Under the coating, two eggs are attached to each other. The inner eggs inside the mass are characterized by a pressed surface due to their attachment to adjacent eggs (Fig. 2C). The adhesive surface has a coating with CM, forming a single homogeneous layer of the CM. Microsphere lumps (Lu) are observed (Fig. 2D). Depending on the amount of CM, the CM may form a multilayer in the form of a lump.

**Fig. 3:**
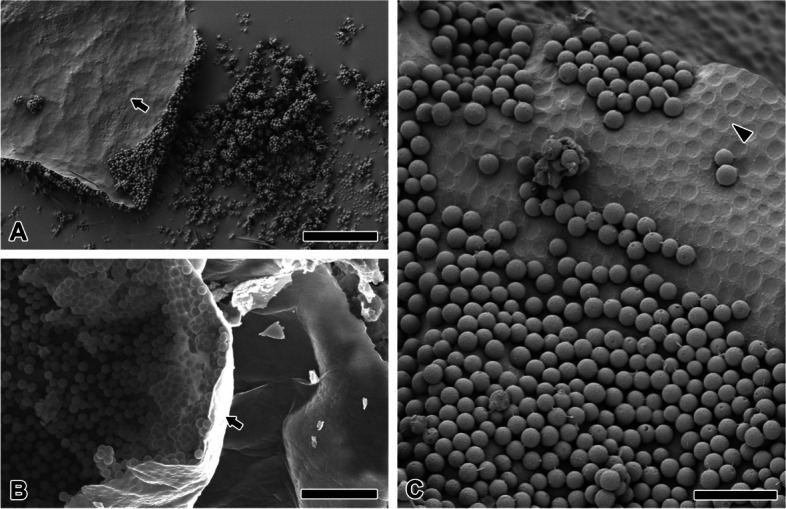
Adhesive chorion surface. **A** Treated with EtOH, the chorion with sticky coating is peeled off. **B** Partially peeled off chorion on the egg shows that microspheres are attached only above the surface. **C** Old chorion has reduced adhesiveness and lost some microspheres. All arrows indicate internal surfaces of the egg chorion and arrowhead indicates external surface of the egg chorion. Each scale bar indicates 50 µm (**A**) and 20 µm (**B**) and 10 µm (**C**), respectively

Treatment with EtOH for 48 h confirm that the CM-coated chorion peeled off on the cover-glass bottom (Fig. 3A). The partially peeled-off chorion shows that CM is attached only above the surface (Fig. 3B). No spherical structure is found under the CM-coated layer. Based on the CM positions and attachment traces, the CM on the surface is generally arranged in a hexagonal pattern. (Fig. 3C). Adhesiveness decreases in the old chorion (over 6 months) surface and CM can fall off. Adhesion traces are clearly observed at the location where the CM has fallen off. The CM was found to have a uniform size distribution, with a diameter of approximately 2.3 µm. The mean diameter of 80 adhesion traces was measured to be 2 µm.

**Fig. 4:**
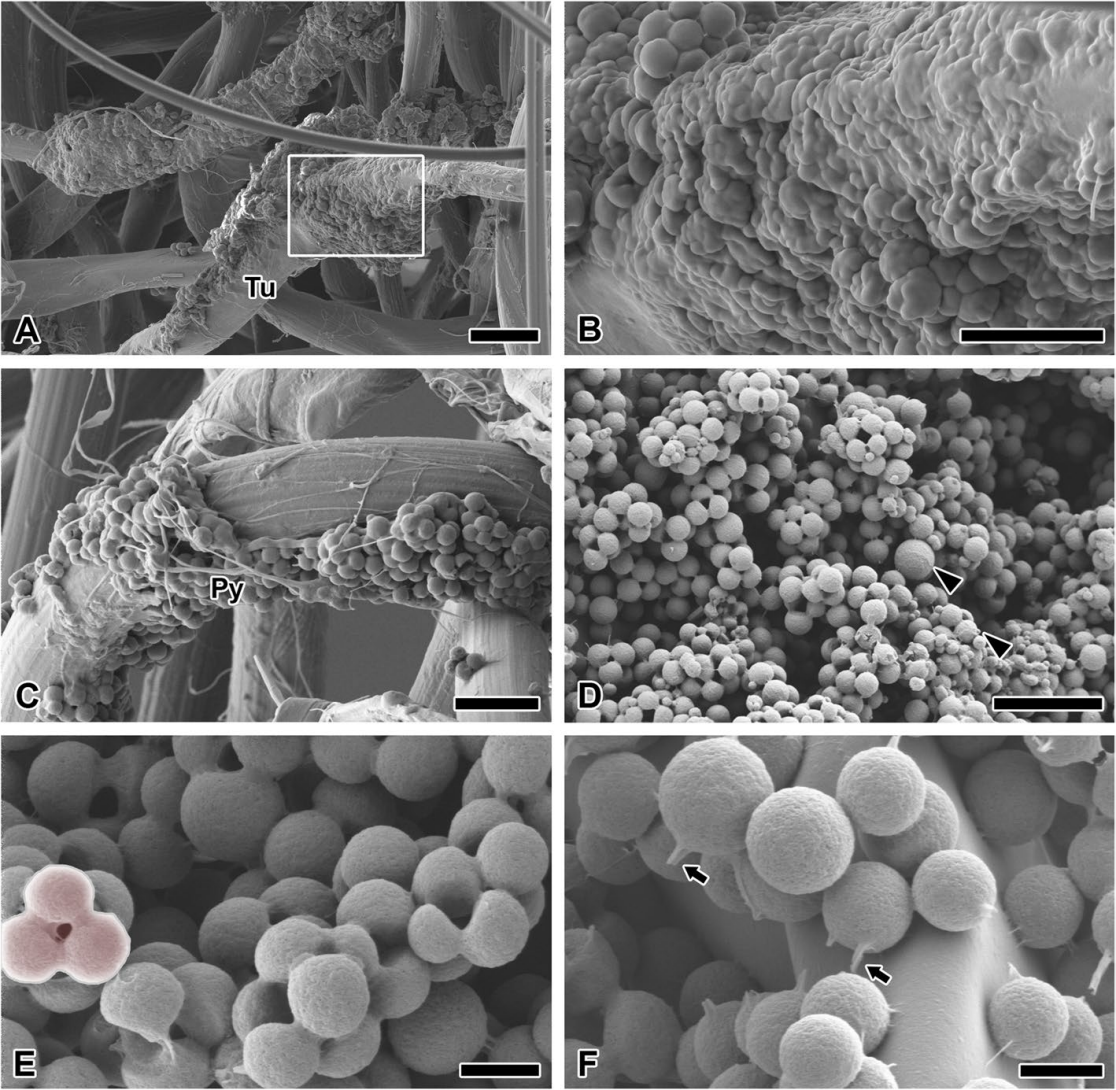
Fine structure of microspheres in eggmass. **A** The oviposition fluid covering tubuliform silk forms a solid platform. **B** Enlarged micrograph of the rectangular zone of Fig. 4A. This solid platform is completely closed rather than a microsphere layer. **C** Pyriform gland-derived glue (Py) adheres to microspheres. **D** The multilayer on the eggmass surface has various sphere diameter size. Arrowheads indicate the largest and smallest spheres. **E** The microspheres on the eggmass surface is predominantly aggregated (outlined range). **F** The monolayer attached to the inner egg mainly shows a ‘papila’ (arrows). Each scale bar indicates 20 µm (**A**), 10 µm (**B**-**D**) and 2 µm (**E**, **F**), respectively

Egg coating can be deposited on the tubuliform silk (Fig. 4A). The solid platform is completely closed and hence does not form an CM layer (Fig. 4B). Meanwhile, a non-porous CM layer is also observed, which maintained its spherical shape (Fig. 4C). Additionally, pyriform gland-derived glue proteins (Py) adhere to CM.

The outside plane of the eggmass has CM of various diameters (Fig. 4D). The CM has diameters that may vary by up to about 39-fold. The CM in the multilayer are predominantly aggregated together, which appears to enable adhesion between CM (Fig. 4E). On the surface of the chorion, a fine papillary projection, along with a broken strand, is mainly observed rather than aggregated form (Fig. 4F). Two or more strands and “papillae” can be observed in one CM unit.

**Fig. 5:**
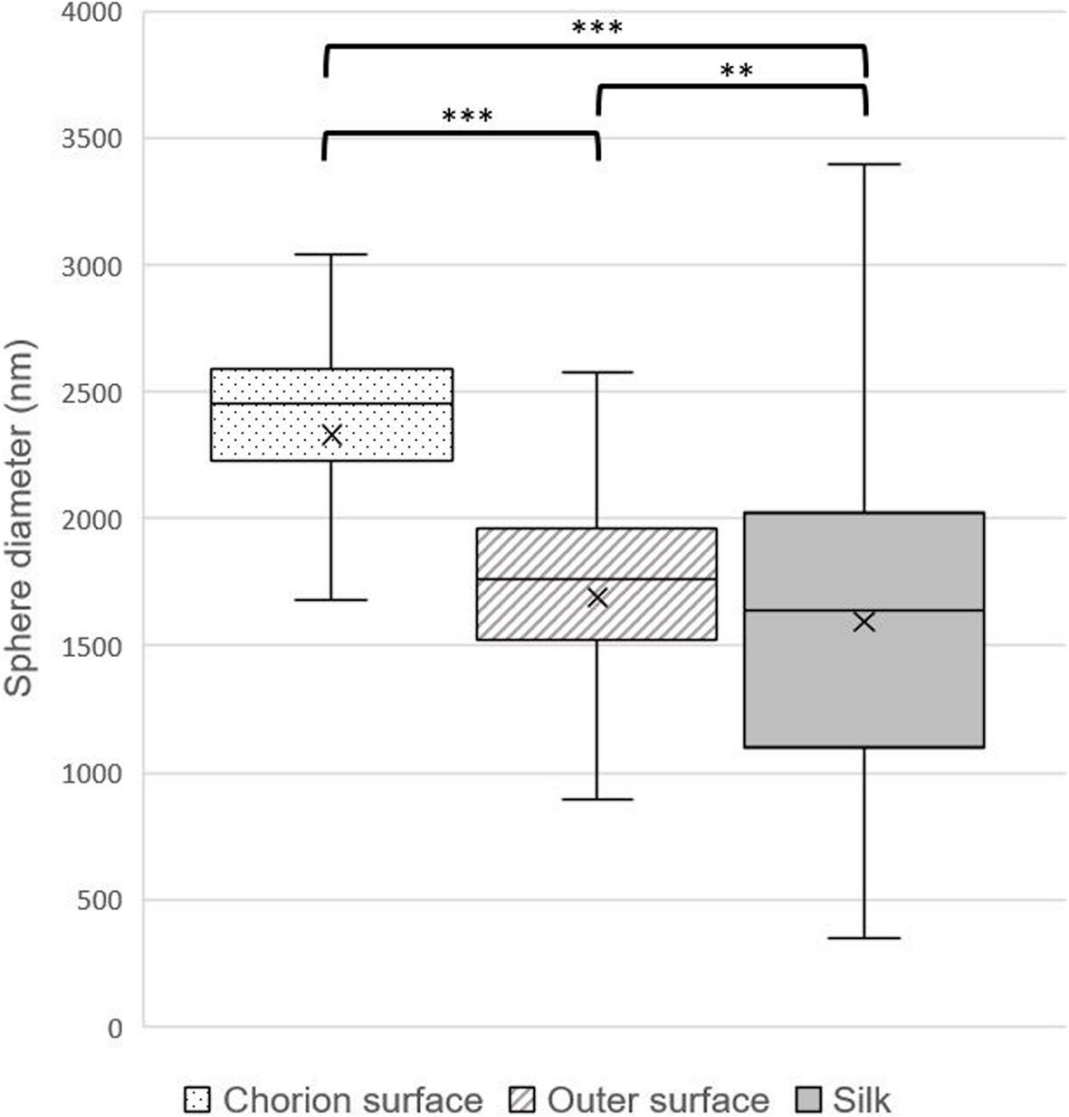
Box plot comparing the diameter distribution in the chorion surface, outer surface, and silk. The median is indicated by a line, while the upper and lower quartiles are enclosed by boxes. Whiskers represent the diameter range. The mean diameter values (2329 nm, 1692 nm, and 1594 nm, respectively) of the 500 spheres calculated are marked with X. Statistical significance is denoted as ** *p* < 0.05 and *** *p* < 0.001"

Next, 500 CM were photographed and their diameters were determined using the freeware imageJ (Schneider et al. 2012). The diameter distributions are plotted in box plots for each location (chorionic surface, outer surface, and silk) (Fig. 5). Their averages diameters are determined to be 2329, 1692, and 1594 nm, respectively (standard deviation: 438.83, 444.67, and 601.79). The boxplot shows that the CM size can vary significantly by location (*p* < 0.005). In particular, the size of the CM inside is significantly larger than that of the outside.

**Fig. 6:**
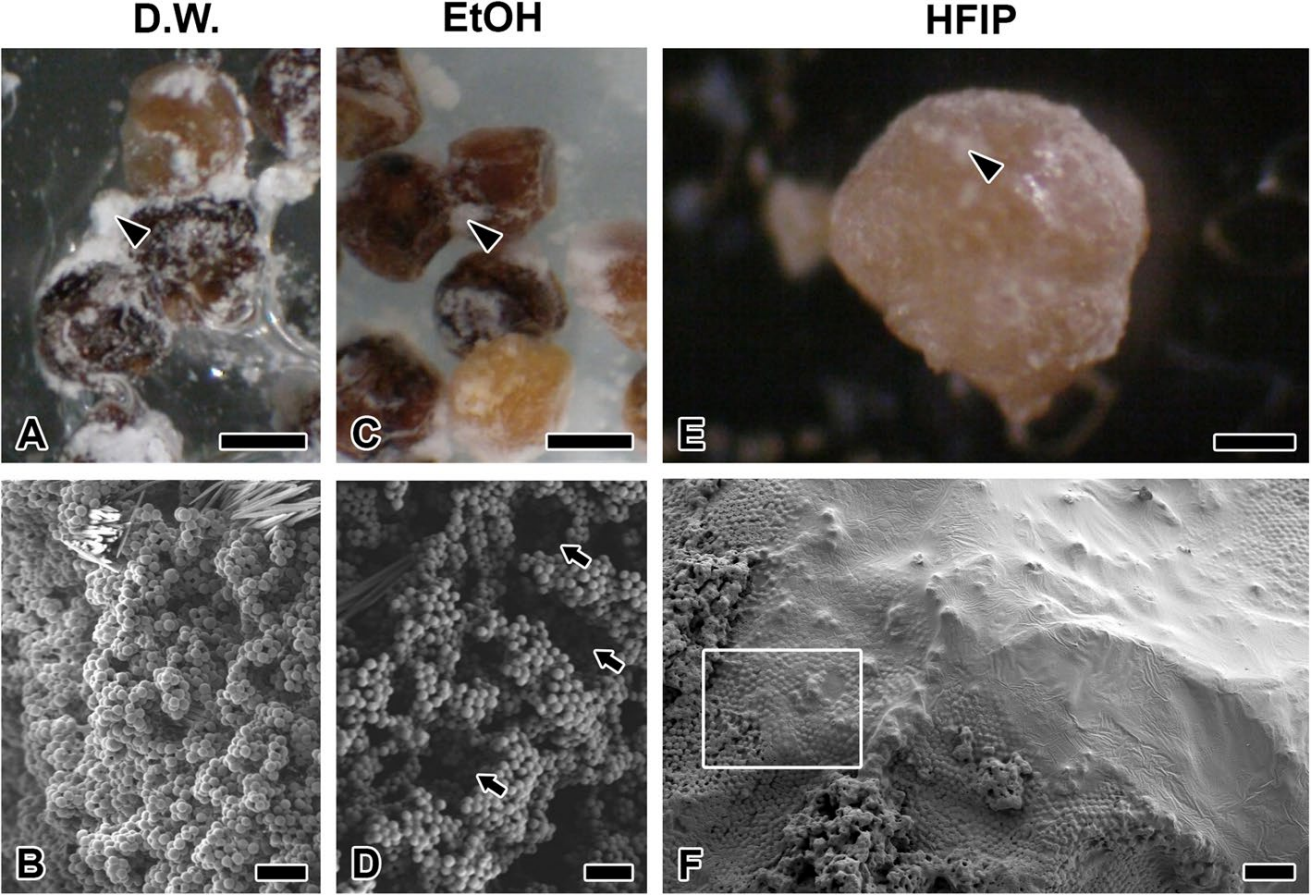
Microsphere structural change by solvent treatment for 48 h. Light microscopic and FESEM (**A, B**: distilled water, **C, D**: EtOH, **E, F**: hexafluoroisopropanol) observation for each solvent treatment shows that microsphere dissolution effect by organic solvent is better than water. Arrowhead indicates lump. **D** The arrow marks the crack where the microsphere is partially dissolved. **F** Most of the spherical structures have been lost with HFIP, and some retain their shape slightly (white zone). Each scale bar indicates 100 µm (**A**, **B**), 50 µm (**C**) and 20 µm (**D**, **E**, **F**), respectively

To confirm the affinity of the CM for various solvents, the eggs were treated with water and anhydrous EtOH and HFIP for 48 h (Fig. 6). The white structure (CM) on the eggs was hardly soluble in water (Fig. 6A) and hence no change is observed on the electron microscope (Fig. 6B). In contrast, the CM partially dissolved in EtOH (Fig. 6C) with considerable amount of white coating detached on the cover glass. The partially dissolved surface of CM appeared as cracked structures aligned on a single “plane” (Fig. 6D). HFIP, a highly volatile organic solvent, can melt the CM layer (Fig. 6E). Most of the spherical structures are lost when treated with HFIP, while some barely retained their shapes (Fig. 6F). The egg surface is found to have a surface that ranges from smooth to a densely CM-coated one.

**Fig. 7:**
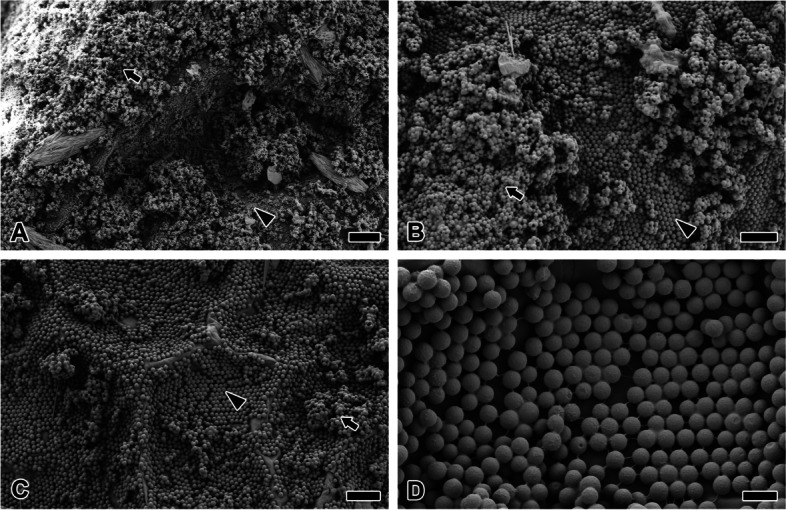
HFIP treatment on eggmass surface within 20 s. **A, B** HFIP not treated eggs shows large portion of multilayer (arrow) surrounding egg. Under the multilayers, single layer (arrowhead) is directly attached to the chorionic surface. **C** Eggs treated with HFIP lost most of the multilayer. **D** The microspheres are arranged with hexagonal regular arrangement. Each scale bar indicates 50 µm (**A**), 20 µm (**B**, **C**) and 5 µm (**D**), respectively

To confirm the intermediate state of the dramatic structural change caused by HFIP, HFIP was applied to the surface for only about 20 s. Outside plane not treated with HFIP comprises a large portion of the multilayer surrounding the egg (Fig. 7A). Under the multilayers, a single layer is directly attached to the chorionic surface (Fig. 7B). In contrast, eggs treated with HFIP lost most of the multilayers (Fig. 7C). Rather than aggregate, strand and papilae structures dominate the CM (Fig. 7D).

**Fig. 8:**
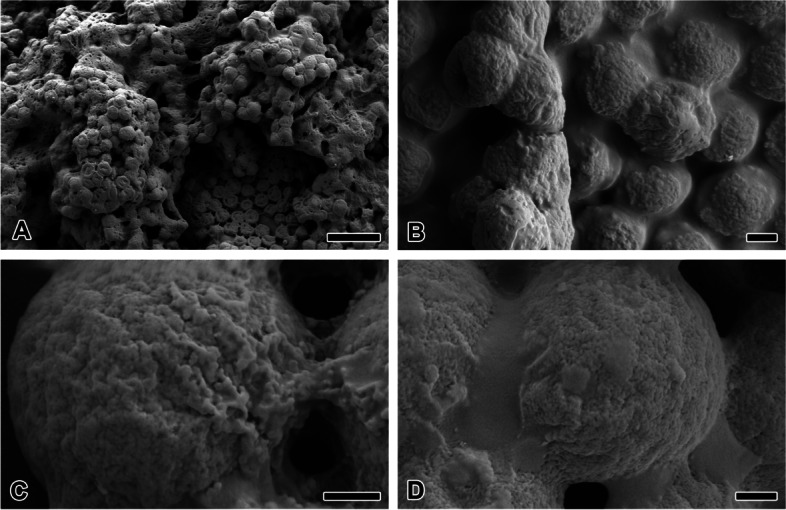
Fine structure of microspheres treated with HFIP. **A** HFIP Immediately dried microspheres were severely distorted in spherical shape and formed a sticky mass with neighboring spheres. **B** HFIP 48 h treated microspheres were shrunken, it appears that the spheres are dissolved in HFIP. **C** Microsphere without any process has severe uneven surface with agglomerated fine particles. **D** HFIP immediately dried microspheres are relatively less granular. Each scale bar indicates 10 µm (**A**), 1 µm (**B**) and 500 nm (**C**, **D**), respectively

The HFIP-treated CM were immediately dried and melted (Fig. 8A). The spherical, but severely distorted CM and a sticky CM mass are mainly observed. The shrinkage of the spherical shape suggested that the CM dissolved in HFIP (Fig. 8B). Fine structure of the eggs not treated with HFIP showed that the CM has a severely uneven surface with agglomerated fine particles (Fig. 8C). The adhesive strand has the same texture as the CM surface, which contains nanoparticles. The CM of the HFIP-treated eggs immediately dried and were relatively less granular. In addition, the surface of the merged strand has a softer texture than that of the control (Fig. 8D).

**Fig. 9:**
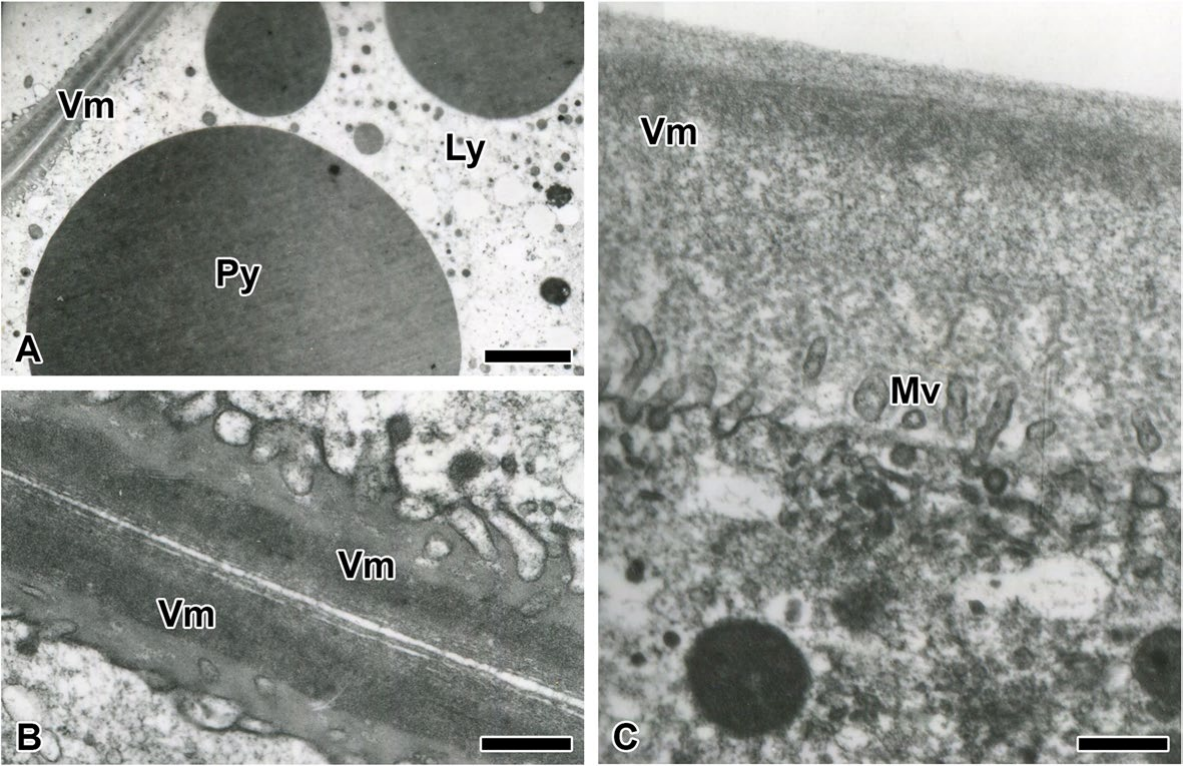
Transmission electron micrograph of the oocyte in *T. clavata.* **A** Each oocyte consists of proteid yolk (Py) and lipid yolk (Ly) including aggregates of fine electron dense granules. The vitelline membranes (Vm) of the oocyte face each other. **B** On the surface or empty space between the vitelline membranes, microspheres or its precursors are not observed. **C** Vitelline membrane is composed of an inner homogeneous electron lucent component and an outer layer of more or less electron dense component. No secretion is observed on the vitelline membrane adjacent to the lumen. Mv: Microvilli. Each scale bar indicates 5 µm (**A**) and 500 nm **B**, **C**

Each mature oocyte is composed of glycogen and yolks of various sizes and types (Fig. 9A). Transmission electron microscopy images shows that the yolk comprised two types of granules: large sized and highly electron-dense yolk protein and low electron-dense yolk lipid. In addition, fine glycogen particles are found to aggregate together and form a high-electron component. The vitelline membrane is formed after the completion of vitellogenesis. Before choriogenesis, the vitelline membrane has an inner homogeneous electron-lucent component, a denser middle layer, and a less dense outer layer (Fig. 9B, C). Unlike the layers in which the vitelline membranes face each other in oocytes (Fig. 9B), the vitelline membrane facing outward has a low electron density (Fig. 9C). Each oocyte has well-developed microvilli along the surface of the egg envelope. CM or its precursors are not identified until chorions are formed.

## Discussion

The orb-web spider, *T. clavata* secretes a large amount of the oviposition fluid while laying eggs. Its eggs are first reddish, which then gradually turn brown. The oviposition fluid surrounds whole eggmass, soon dries to a white CM. This milky coating consists of a high-density CM surrounding the chorion. In the genus *Trichonephila*, the CM coating is thought to be significantly thicker than that in other reported spider taxa (Humphrey 1987, 1995). Therefore, CM exhibits minor morphological differences depending on their location. This study describes the structural variations and features of CM in *T. clavata*.

The CM of *Araneus* (Araneidae), was observed to have an average diameter of 4.6 µm or higher, while most families of Araneae exhibit a CM of around 1 µm (Grim and Slobodchikoff 1982; Humphrey 1983). At the level of Araneidae, the average size of the CM ranges from 1.16 µm (*Argiope aetherea*) to 3.55 µm (*Archemorus simsoni*) (Humphrey 1987). In *Trichonephila*, *Trichonephila edulis* was found to have a uniform CM diameter with an average of 1.6 µm (Humphrey 1995).

This study demonstrates that the CM of *T. clavata* exhibits a consistent size distribution of 2.3 µm, limited to the chorionic surface within the eggmass. However, average diameter of the CM exposed on the outer eggmass was approximately 1.7 µm. The reason for the existence of such spherical size variation is not yet known, but it seems to be a common phenomenon during the production and secretion of granules. This is because mature granules stored within secretory cells are first deposited on the chorionic surface, and immature granules subsequently accumulate on the outer surface.

Alternatively, the size difference of CM may have been caused by the difference in exposure to external environment due to the thick CM layer. A diverse range of CM with varying diameters was found on the silk covering the eggmass, but their varied forms make it difficult to determine when they were attached to the silk.

Adhesive structures formed between CM can be categorized into three stages: aggregates, strands, and papillae. Aggregates refer to clusters of CM, while strands connect individual CM unit, and papillae are short projectional structure. The “papilla” was also firstly reported by Humphrey (1995). These three structures are believed to form aggregated CM clumps that elongate because of external factors (e.g., diffusion of secretions or airflow) and form a strand, which eventually breaks off to become papillae.

These segmental adhesions are composed of a substance, which is different from the spherical scaffolds that maintains its shape for a long time. According to Witaliński and Žuwała (1981) and Makover et al. (2019), the oviposition fluid contains mucus and low-molecular-weight proteins. The low-molecular-weight proteins indicates the basic spherical units of CM, while mucus forms the adhesion between the units. Makover et al. (2019) reported that the CM in the *Latrodectus geometricus* egg is insoluble, although it is superhydrophilic with a water contact angle of <10°. Because CM itself is found to be insoluble, the superhydrophilic layer on the CM is probably made of mucus secretions.

If CM acquires adhesive ability through the mucus components, it can explain the stronger adhesion on the chorionic surface than between CM. The chorion exposed to air for a long period (over 3 months) shows poor adhesion. The CM that do not adhere directly to the surface are more likely to fall off. Nevertheless, the diameter of the fall-off trace is comparable to 87% of the CM diameter. Therefore, approximately 34% of the CM is in contact with the chorionic surface. These quantitative measures could potentially serve as indicators for explaining the differences in adhesive properties. What is certain is that CM themselves do not possess any adhesive properties.

CM or its precursors is not observed in the vitelline membrane during oogenesis. In addition, the egg does not contain any tissues or cells secreting a microgranular structure. Therefore, this result suggests that the CM does not derived from both vitellogenenic and choriogenetic processes. Morishita et al. (2003) also reported that the eggs of *Loxosceles intermedia* become granular during the transition of the oocytes through the oviduct and the uterus. Therefore, it is presumed that the CM components are separately secreted from the exocrine ducts or secretory cells present in the oviduct and the uterus.

Regarding CM as an ecophysiological parental investment, it is necessary to compare the function of eggcase silk, as same means. Spiders that overwinter in the cocoon protect the larva from desiccation, while those that do not overwinter provide no protection to the eggs from transpiration (Hieber 1992). According to Austin (1984), the cocoons of sac spiders *Clubiona robusta* (Clubionidae) have a higher humidity than the surrounding air and prevent the eggs from drying out. Schaefer (1976) experimentally demonstrated that the eggs of *Floronia bucculenta* (Linyphiidae) survive in the cocoon for 68 days at 32% relative humidity and 5°C. On the other hands, he also reported the eggs dry out in 37 days without the eggcase.

Austin and Anderson (1978) showed that the cocoons of orb-web spider, *Trichonephila edulis*, which is a non-overwintering species, offer no protection to the eggs from desiccation. However, since *T. clavata* makes loosely silken eggpad, more precise humidity control is required to overwinter. It is expected that this function will be complemented by thick CM layers. Alternatively, a closed solid platform, presumed to be a mixture of silk proteins and non-silk CM, may be associated with protection from desiccation.

The CM of Lycosidae, which builds compact eggcase, are attached to chorionic surface without forming any distinct CM layers (Grim and Slobodchikoff 1982). The microparticle structure of the exochorion can be found in several insects. Witaliński (1993) suggested that the exochorion granular structure in mites (Sarcoptiformes) such as *Aleuroglyphus ovatus*, *Tyrophagus perniciosus*, and *Notoedres cati* functions as an adhesive layer that fixes the eggs to the host epidermis. Dragonflies such as *Brachythemis lacustris* and *Tholymis tillarga* (Libellulidae) contain a trabeculate endo chorion and an exochorion perforated by aeropyles (Miller 1987).

Wetting the exochorion at the oviposition causes it to become sticky and to attach firmly to the substrate. In the case of *T. clavata*, the abundant outer layer of CM in the eggmass serves as a barrier that prevents the eggs from scattering, much like a compact eggcase. Conversely, if an "eggcase" is capable of sufficiently preventing the dispersion of eggs, there may be no need for a large secretion of oviposition fluid.

Makover et al. (2019) also reported that the CM of eggs in *Latrodectus geometricus* had antibacterial effects. Esteves et al. (2009) described an antimicrobial surface of the eggs of the *Rhipicephalus* tick (subgenus: *Boophilus*). Similar findings were reported by Yu et al. (2012) and Zimmer et al. (2013) for ticks in *Amblyomma hebraeum* and *Rhipicephalus microplus*, respectively. Nelson et al. (1999, 2000) described a waxy, fluffy material produced by whitefly females into which they lay eggs.

The material, according to the authors, camouflages the eggs, thus protecting it against predators. In vertebrates, D'Alba et al. (2013) showed that the surface topography of brush-turkey *Alectura lathami* eggs, determined by the presence of nanoscale spheres composed of calcium phosphate, renders the eggs hydrophobic, decreases bacterial attachment, and is most likely the major component preventing trans-shell penetration. Although the morphology is different, these egg surface microstructures are confirmed to be the result of convergent evolution that function in protecting eggs.

The egg-coating function of the CM in spiders has been anticipated to be similar to that of cocoon silk. However, the morphology and characteristics of CM have remained understudied for a long time. The results of this study strengthen the premise that the CM of *T. clavata* is formed by the combination of spherical insoluble proteineous units and adhesive mucus component. On the other hand, it is proposed that CM and eggcase silk have a high complementarity in their functions, suggesting that they have evolved to have close interactions.

This idea could also be applicable to further biological studies on the egg secretion of silk-producing arthropods. It would be interesting to investigate whether there is a correlation between the amount of CM secretion and the average winter climate in the area, in relation to the proliferation and northward expansion of *T. clavata* (Chuang et al. 2023), which has been introduced into the United States. The egg mass of the golden orb web spider, *T. clavipes*, which is closely related to *T. clavata*, is surrounded by a large amount of white coating. However, unlike the restricted distribution of *T. clavipes* in the southern regions of North America, the invasive species *T. clavata* is rapidly spreading to central areas (Davis and Frick 2022).

It has been revealed that *T. clavata* has a greater cold resistance at the individual level compared to *T. clavipes*, which suggests its potential to survive even in the average temperatures of northern United States. Although the differences in cold adaptation in spiders can be explained by the presence or absence of the heat-shock hatching (Schaefer 1987), it would be interesting to explore the relationship between temperature and CM through comparative analysis between the CMs of the two species.

## Conclusions

We examined the fine structural characteristics of the CM coating chorionic surface of *T. clavata*. Morphological differences of CM were observed using scanning electron microscope. Although the CM exhibits an uneven size distribution in outer egg mass, the chorionic surface is evenly covered with a single layer with a diameter of 2.3 µm approximately. The surface structure of aggregated CM shows short papillary projections demonstrating segmental adhesion of mucous components. CM is insoluble in water but partially soluble in anhydrous ethanol, and its spherical structure is completely decomposed by HFIP, a strong organic solvent. Since our fine structural observation of the ovarian developmental process clearly show that CM is not derived from vitellogenic or choriogenetic processes, the CM adhesive coatings during ovipositional process appears to be equivalent to cocoon silk for various protective functions in silken eggcase.

## Data Availability

Materials described in the manuscript, including all relevant raw data, will be freely available to any scientist wishing to use them for non-commercial purposes.

## Abbreviations

HFIP: Hexafluoroisopropanol
CM: Chorionic microspheres

## References

[CR1] Chuang A, Deitsch JF, Nelsen DR, Sitvarin MI, Coyle DR (2023). The Jorō spider (Trichonephila clavata) in the southeastern U.S.: an opportunity for research and a call for reasonable journalism. Biol. Invasions..

[CR2] Austin AD, Anderson DT (1978). Reproduction and development of the spider *Nephila edulis* (Koch) (Araneidae: Araneae). Aust. J. Zool..

[CR3] Austin AD (1984). Life history of Clubiona robusta L. Koch and related species (Araneae, Clubionidae) in South Australia. J. Arachnol..

[CR4] Austin AD (1985). The function of spider egg sacs in relation to parasitoids and predators, with special reference to the Australian fauna. J. Nat. Hist..

[CR5] Davis AK, Frick BL (2022). Physiological evaluation of newly invasive jorō spiders (*Trichonephila clavata*) in the southeastern USA compared to their naturalized cousin. Trichonephila Clavipes. Physiol. Entomol..

[CR6] Schneider CA, Rasband WS, Eliceiri KW (2012). NIH Image to ImageJ: 25 years of image analysis. Nat. Methods.

[CR7] Hieber CS (1985). The “insulation” layer in the cocoons of *Argiope aurantia* (Araneae: Araneidae). J. Therm. Biol..

[CR8] Hieber CS (1992). The role of spider cocoons in controlling desiccation. Oecologia.

[CR9] Nelson DR, Fatland CL, Buckner JS, Freeman TP (1999). External lipids of adults of the giant whitefly Aleurodicus dugesii. Comp. Biochem. Physiol. B, Biochem. Mol. Biol..

[CR10] Nelson DR, Freeman TP, Buckner JS (2000). Waxes and lipids associated with the external waxy structures of nymphs and pupae of the giant whitefly. Aleurodicus dugesii. Comp. Biochem. Physiol. B, Biochem. Mol. Biol..

[CR11] Conti E, Costa G, Marletta A, Viscuso R, Vitale DG (2015). The chorion of eggs in a *Namibian Ariadna* species (Araneae: Segestriidae): morphological and SEM analyses. J. Arachnol..

[CR12] Esteves E, Fogaça AC, Maldonado R, Silva FD, Manso PPA, Pelajo-Machado M, Valle D, Daffre S (2009). Antimicrobial activity in the tick *Rhipicephalus* (Boophilus) microplus eggs: Cellular localization and temporal expression of microplusin during oogenesis and embryogenesis. Dev. Comp. Immunol..

[CR13] Hoebeke ER, Huffmaster W, Freeman BJ (2015). *Nephila clavata* L koch, the Joro spider of East Asia, newly recorded from North America (Araneae: Nephilidae). PeerJ.

[CR14] Barrantes G, Sandoval L, Sánchez-Quirós C, Bitton PP, Doucet SM (2013). Variation and possible function of egg sac coloration in spiders. J. Arachnol..

[CR15] Grim JN, Slobodchikoff CN (1978). Chorion surface features of some spider eggs. Pan-Pac. Entomol..

[CR16] Grim JN, Slobodchikoff CN (1982). Spider egg chorion sphere size and density. Ann. Entomol. Soc. Amer..

[CR17] Schultz JW (1987). The origin of the spinning apparatus in spiders. Biol. Rev..

[CR18] Zimmer KR, Macedo AJ, Nicastro GG, Baldini RL, Termignoni C (2013). Egg wax from the cattle tick *Rhipicephalus* (Boophilus) *microplus* inhibits *Pseudomonas aeruginosa* biofilm. Ticks. Tick. Borne. Dis..

[CR19] D'Alba L, Jones DN, Eliason C, Badawy HT, Shawkey MD (2013). Antimicrobial properties of a nanostructured eggshell from a compost-nesting bird. J. Exp. Biol..

[CR20] Kuntner M, Hamilton CA, Cheng R-C, Gregorič M, Lupše N, Lokovšek T, Lemmon EM, Lemmon AR, Agnarsson I, Coddington JA, Bond JE (2018). Golden orbweavers ignore biological rules: phylogenomic and comparative analyses unravel a complex evolution of sexual size dimorphism. Syst. Biol..

[CR21] Schaefer M (1976). An analysis of diapause and resistance in the egg stage of *Floronia bucculenta* (Araneida: Linyphiidae). Oecologia.

[CR22] Schaefer M, Nentwig W (1987). Life cycles and diapause. Ecophysiology of Spiders.

[CR23] Moon MJ, Tillinghast EK (2020). Molt-related changes in the ampullate silk gland of the barn spider *Araneus cavaticus*. Animal Cells Syst..

[CR24] Hadley NF (1978). Cuticular permeability and lipid composition of the black widow spider. Latrodectus Hesperus. J. Comp. Physiol. B.

[CR25] Yu P, Liu X, Hayashi F (2022). Functions of egg-coating substances secreted by female accessory glands in alderflies, fishflies and dobsonflies (Megaloptera). Insects.

[CR26] Miller PL (1987). Oviposition behaviour and eggshell structure in some libellulid dragonflies, with particular reference to *Brachythemis lacustris* (Kirby) and *Orthetrum coerulescens* (Fabricius)(Anisoptera). Odonatologica.

[CR27] Morishita R, Aparecida Ferreira S, Santiago Filha A, DitzelFaraco C (2003). Studies on oogenesis and oviposition in the brown spider *Loxosceles intermedia* (Araneae: Sicariidae). Anat. Rec..

[CR28] Foelix RF (2011). Biology of Spiders, 3edn.

[CR29] Makover V, Ronen Z, Lubin Y, Khalaila I (2019). Eggshell spheres protect brown widow spider (*Latrodectus geometricus*) eggs from bacterial infection. J. Royal Soc. Int..

[CR30] Humphreys WF (1995). Chorion surface features of chelicerate eggs. Rec. West. Aust. Mus. Suppl..

[CR31] Humphreys WF (1987). The accoutrements of spiders' eggs (Araneae) with an exploration of their functional importance. Zool. J. Linn. Soc..

[CR32] Humphreys WF (1983). The surface of spiders' eggs. J. Zool..

[CR33] Witaliński W (1993). Egg shells in mites: vitelline envelope and chorion in Acaridida (Acari). Exp. Appl. Acarol..

[CR34] Witaliński W, Žuwała K (1981). Ultrastructural studies of egg envelopes in harvestmen (Chelicerata, Opiliones). Int. J. Invetebr. Reprod..

[CR35] Sun Y, Lee SM, Ku BJ, Moon MJ (2023). Fine structural aspects on the web glue production in the golden orb-web spider *Trichonephila clavata*. Animal Cells Syst..

[CR36] Sun Y, Lee SM, Ku BJ, Park EA, Moon MJ (2021). Capture silk scaffold production in the cribellar web spider. Appl. Microsc..

[CR37] Yu Z, Thomson EL, Liu J, Dennis JJ, Jacobs RL, Kaufman WR (2012). Antimicrobial activity in the egg wax of the tick *Amblyomma hebraeum* (Acari: Ixodidae) is associated with free fatty acids C16: 1 and C18: 2. Exp. Appl. Acarol..

